# Medicinal use of fauna by a traditional community in the Brazilian Amazonia

**DOI:** 10.1186/1746-4269-8-37

**Published:** 2012-09-27

**Authors:** Flávio B Barros, Susana AM Varela, Henrique M Pereira, Luís Vicente

**Affiliations:** 1 Universidade Federal do Pará, Núcleo de Ciências Agrárias e Desenvolvimento Rural (NCADR), Programas de Pós-Graduação em Agriculturas Amazônicas (NCADR) e Antropologia (IFCH), Cidade Universitária José da Silveira Netto, Rua Augusto Corrêa, N° 1, Guamá, Belém, 66075-110, Pará, Brazil; 2 Centro de Biologia Ambiental (CBA), Departamento de Biologia Animal, Faculdade de Ciências da Universidade de Lisboa,, Universidade de Lisboa, Edifício C2, Campo Grande, Lisboa, 1749-016, Portugal; 3 Centre for Environmental and Marine Studies (CESAM), Departamento de Biologia Animal, Faculdade de Ciências da Universidade de Lisboa, C2-P3 Campo Grande, Lisboa, 1794-016, Portugal

**Keywords:** Ethnozoology, Ethnomedicine, Zootherapy, Brazilian Amazonia, Species conservation, Use value (UV) index, Medicinal applications value (MAV) index

## Abstract

**Background:**

Zootherapy inventories are important as they contribute to the world documentation of the prevalence, importance and diversity of the medicinal use of animals in traditional human communities. The present study aims to contribute with a more valuable example of the zootherapy practices of a traditional community in the Brazilian Amazonia – the “Riozinho do Anfrísio” Extractive Reserve, in Northern Brazil.

**Methods:**

We used the methods of participant observation and semi-structured interviews, applied to 25 informants. We employed the combined properties of two indices to measure the medicinal importance of each cited species to the studied community, as well as their versatility in the treatment of diseases: the well known Use Value (UV) and the Medicinal Applications Value (MAV) that we developed.

**Results:**

We recorded 31 species of medicinal animals from six taxonomic categories, seven of which are new to science. The species are used for the treatment of 28 diseases and one species is used as an amulet against snakebites. The five species with the highest UV indices are the most popular and valued by the studied community. Their contrasting MAV indices indicate that they have different therapeutic properties: specific (used for the treatment of few diseases; low versatility) and all-purpose (several diseases; high versatility). Similarly, the most cited diseases were also those that could be treated with a larger number of animal species. Ten species are listed in the CITES appendices and 21 are present in the IUCN Red List. The knowledge about the medicinal use of the local fauna is distributed evenly among the different age groups of the informants.

**Conclusions:**

This study shows that the local fauna represents an important medicinal resource for the inhabitants of the protected area. The combined use of the UV and MAV indices allowed identifying the species with the highest therapeutic potential. This type of information about a species may be of interest to pharmacological research, and is crucial to its conservation, since it helps signaling the species that may undergo higher hunting pressures. Data on zootherapy can also be of interesting to ecologists by contributing to indicators of local biodiversity richness.

## Background

In the field of traditional medicine, animals and their body parts and/or the derived products of their metabolism have long been used as a source of medicinal drugs in different parts of the world and in the most distinct human cultures [1-10]. Zootherapy is raising increasing attention among scientists and research institutions from various countries, since it is part of the human cultural diversity and represents an important alternative to urban modern medicine, or even the only alternative, along with the medicinal plants, for the most economically deprived communities [6,11,12]. Traditional medicine is also a potential source of knowledge to the discovery of new drugs to the modern medicine, though laboratory studies about the chemical and biological properties of these products, which would validate their effectiveness in the treatment of diseases and consequently their relevance to human health, are surprisingly scarce and still preliminary [13]. In zootherapy, researchers are additionally concerned in discerning the risks that some of those products may pose to the communities that use them due to the poor conditions often involved in their preparation or storage [4,8,11]. Ultimately, zootherapy has raised concerns about its impacts to biodiversity, as some of the most valued species are also threatened with extinction [4,12-14]. One classic example is the use and wide commercialization of the body parts of species such as the bear, the tiger and the Asian rhinoceros in traditional Chinese medicine [4]. In this context, ethnobiological studies on the zootherapy practices of traditional communities around the world are of the most relevance, as they help establishing a global information bank of the animal species most used in traditional medicine, highlighting their ecological and cultural value, but also the species that may undergo higher pressures [6].

Brazil, with more than 8 million km^2^ of land area, has a rich cultural and biological diversity, which in the context of traditional medicine provides a countless amount of alternative drugs for the treatment of various diseases. Therefore, several studies have been developed in Brazil on zootherapy and ethnozoology, a trend that has been consistently growing in the last decade [15], particularly in the Northeast region, where the medicinal use of animals has been part of the traditional communities’ secular practices (e.g., [16-22]). Costa Neto and Alves [23] compiled the use of 326 animal species, mostly consisting of fish (92 spp.), mammals (65 spp.), reptiles (44 spp.) and birds (47 spp.), which are claimed to treat diseases such as asthma, rheumatism, muscular pain, cancer and male impotence, among others. The Brazilian traditional medicine is also strongly linked to a religious and belief system, known locally as “simpatias” (sympathies) [6], where the animals can be used, for example, against evil eye or to avert negative energies.

In the Brazilian Amazonia, however, home of many indigenous and non-indigenous traditional communities, only a few studies have focused on zootherapy practices, namely the works developed by Branch and Silva [24], Figueiredo [25], Pinto and Maduro [26], Alves and Santana [27], Alves and Rosa [28], Rodrigues [29], Alves and Rosa [30], Ribeiro et al. [31], Alves and Rosa [32], and Silva [33]. These works cataloged about 140 animal species with medicinal use in the Brazilian Amazonia, which represents 43% of the species when compared to the total number recorded in Brazil. Aiming to contribute with one more valuable example of zootherapy in Amazonia, here we describe the various forms of medicinal use of several animal species by a traditional riverine community living at the “Riozinho do Anfrísio” Extractive Reserve (Pará, Northern Brazil). This is the first study of ethnomedicine made in this protected area and one of the few in Northern Brazil (see Alves and Souto [15]). Following Alves at al. [6], we wish to contribute to an information bank of animal derived remedies in Amazonia that would help documenting the prevalence, importance and diversity of these practices in the indigenous and traditional communities where they occur. Understanding this will not only help to preserve the culture and natural habitats of these communities, as it will also signal the animal species most used for medicinal purposes, a kind of information that is crucial to our knowledge of the species and to their conservation, especially to the taxa which status indicates already some level of concern. For a more comprehensive evaluation of the medicinal importance of each animal species to the “Riozinho do Anfrísio” community, we employed two indices: the well known “Use Value” (UV) index [34-36] and the “Medicinal Applications Value” (MAV) index that we developed.

## Methods

### Study site

**Figure 1:**
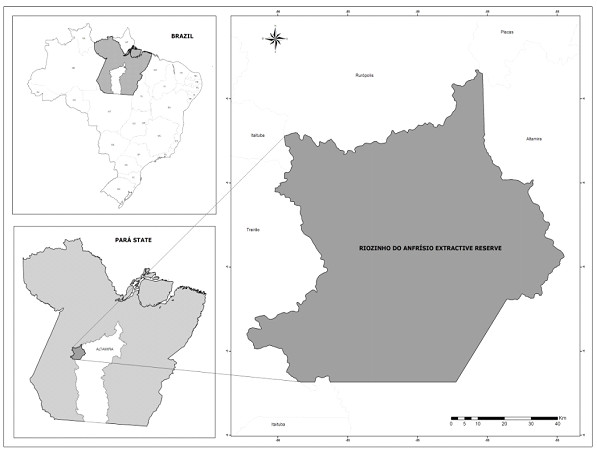
**Map of the studyarea.** Map of the “Riozinho do Anfrísio” Extractive Reserve, State of Pará, Brazil.

The “Riozinho do Anfrísio” Extractive Reserve (54°39’18.28”W, 4°45'33.98”S), a Protected Area (PA) of Sustainable Use with 736 340 ha, is located in Altamira municipality, State of Pará, Northern Brazil (see Figure 1), in the Brazilian Amazonia rainforest, where the average annual temperature and rainfall are 27°C and 1885 mm, respectively [37]. The riverine community of the PA consists of 55 families totaling 300 inhabitants, mostly children and youth. This community is the result of miscegenation between local indigenous peoples, natives from other Amazonian regions and migrants from the North-eastern Brazil, who came to this area at the time of the caoutchouc exploitation, between the end of the nineteenth and early twentieth centuries. When the caoutchouc exploitation ended, most migrants left the area. New immigration reduced accentually after that time and became forbidden in 2004, after the creation of the PA. The main economic activities of this riverine community are fishing, gathering of Brazil-nuts *Bertholletia excelsa*, hunting and farming (shifting cultivation of subsistence). The residents have no medical services, electricity or sanitation. The Reserve is located about 400 km from the urban center of Altamira. Travelling is made mainly by boat through the rivers Xingu and Iriri. Depending on the season (winter or summer) and on the type of boat used (slower collective boats for people and merchandises or faster individual launches, known in the region as “voadeiras”), the time taken from Altamira to the reserve varies between two to eight days.

### Data collection

We travelled eight times to the “Riozinho do Anfrísio” Extractive Reserve, in the period between June 2008 and March 2010. We used semi-structured interviews [38] and participant observations [39,40]. From the 55 families of the PA, 25 were selected based on their location (accessibility from the river), on whether they agreed to participate in the interviews and on their presence by the time of our visit. For the semi-structured interviews component of our methodology, we talked to one person per family, the household head, regardless of their sex or age. They were 24 men and 1 woman, aged between 18 and 83 years old. We directly asked questions about the kinds of diseases and other health problems that were affecting the community, what animal species were used to treat them, which body parts were collected and which methods of preparation were employed. For the participant observation component, we collected data by travelling with the informants in their hunting and agricultural activities, or participated in the family meals, and recorded information on an animal whenever it was mentioned by the informants or by their family members as medicine. Participant observations were also used to identify, in the field, the animal species cited by the informants during the interviews. In summary, the final data consist of information collected among the 25 informants during direct enquiries and in more informal situations where other people could also have been present. Given the difficulties in travelling within the reserve, each informant and its family was visited, interviewed and followed during participant observations in only one of the eight expeditions to the reserve. On average, we stayed with each family for a period of one week.

After the expeditions, we identified the animal species cited by the informants through photographs, descriptions and vernacular names and with the help of taxonomists. Their conservation status was verified with the CITES appendices (2010) [41] and the IUCN Red List of Threatened Species (2010) [42]. We conducted the study with the written consent of the local community and with the proper authorization from the Chico Mendes Biodiversity Conservation Institute (ICMBio) and from the Brazilian Institute of Environment and Natural Resources (IBAMA), licenses 13259–1 and 20477–1.

### Data analysis

For a quantitative analysis of the data we used the semi-structured interviews only. Participant observations, because they come from a different sample and were collected opportunistically by observing the informants in their daily activities, they were used only qualitatively to help contextualizing the results. Because the informants are descendants of local indigenous peoples, all information gathered from the interviews was considered representative of the local culture – despite a certain level of miscegenation with immigrants from the time of the caoutchouc exploitation (as explained above) – and included in the analyses, even if it has been supplied by only one person [43,44].

We applied the non-parametric Spearman Rank Order Correlation coefficient to assess if there was a significant relationship between the number of species cited and the age of the informants. To measure the degree of medicinal importance of each species cited by the informants, we employed two indices:

i. the index of “Use Value” (UV) [34-36] that takes into account the number of informants citing each species for medicinal purposes; and

ii. the index of “Medicinal Applications Value” (MAV) that we developed and that takes into account the number of use-categories of diseases and other health problems cited by the informants for each species.

We applied the Spearman Rank Order Correlation Coefficient to assess if there was a significant relationship between the number of informants (UV) and the number of use-categories (MAV) for each species. Finally, and from the point of view of the diseases cited, we applied again the Spearman correlation coefficient to determine if there was a significant relationship between the number of times a disease was cited by the informants and the number of species used to treat that disease. Correlations were tested with *Statistica 8.0* (Stat Soft, Inc. 1987–2007).

Classically, the UV of a species *s* is calculated as the number of medicinal uses (use-reports) an informant *i* knows for a species *s* (*UV*_*is*_), divided by the total number of informants (*N*) [34,35]:

$$UV_{s}=\frac{\sum_{i=1}^{N}UV_{\mathrm{is}}}{N}$$

In a simplified version, the UV of a species depends exclusively on the number of informants citing that species [36]. It is calculated by assigning *UV*_*is*_ = 1 for any species with non-zero number of uses by informant *i*, being the proportion of incidence or popularity of the species among informants. It is, in other words, the measure of the spread of the knowledge about the species in the studied community, revealing at the same time the level of agreement about that species among the informants. This normalized version of the UV index thus varies from 0 to 1. When low or close to zero, it means that the knowledge about the species is not widespread among the community. When close to 1, it means that the species is known in the community by almost all informants.

The Medicinal Applications Value (MAV) index shows the proportion of medicinal use-categories (diseases or other health problems) that the informants claim to be treatable by a certain animal species. It is the measure of the versatility of the medicinal uses of the species. To facilitate future comparisons between ethnomedicinal studies from different traditional communities around the world, we defined as use-categories the categories of diseases described by the International Statistical Classification of Diseases and Related Health Problems [45] of the World Health Organization, instead of the exact number of diseases cited by the informants of a specific community. Accordingly, the MAV index of a species *s* was calculated as the number of ICD categories that informants claim to be treatable by the species *s* (*D*_*s*_), dividing by the total number of ICD categories defined by the World Health Organization (*D*_*t*_), which were 20 categories on its last update [45] (see Table 1):

$$MAV_{s}=\frac{D_{s}}{D_{t}}$$

**Table 1:** **The 20 disease categories of the World Health Organization**[45]

**International Statistical Classification ofDiseases and Related HealthProblems (ICD, 2007)**		**Cited diseases**
1	Certain infectious and parasitics diseases	Erysipelas
		Hair lice*
2	Neoplasms	Tumor
3	Diseases of the blood and blood-forming organs and certain disorders involving the immune mechanism	none
4	Endocrine, nutritional and metabolic diseases	Diabetes*
		Lack of appetite in children
5	Mental and behavioral disorders	none
6	Diseases of the nervous system	Spasm in children
7	Diseases of the eye and adnexa	Headache
7	Diseases of the ear and mastoid process	Conjunctivitis*
8	Diseases of the circulatory system	Earache
9	Diseases of the respiratory system	Stroke
10		Pneumonia
		Asthma
		Cold
11	Diseases of the digestive system	Indigestion
		Toothache*
12	Diseases of the skin and subcutaneous tissue	Callus on feet*
13	Diseases of the musculoskeletal system and connective tissue	Muscular pain
		Rheumatism
		Child with walking difficulties
		Hernia
14	Diseases of the genitourinary system	Menstrual pain
		Male sexual incapacity
15	Pregnancy, childbirth and puerperium	none
16	Certain conditions originating in the perinatal period	none
17	Congenital malformations, deformations and chromosomal abnormalities	none
18	Symptoms, signs and abnormal clinical and laboratory findings, not elsewhere classified	Hiccup*
		Bleeding
19	Injury, poisoning and certain other consequences of external causes	Snakebite
		Ray stinging*
		Wounds
		Extraction of wood and thorn
20	External causes of morbidity and mortality	none

Legend: In the “Cited Diseases” column it is listed the 28 diseases and other health problems that the “Riozinho do Anfrísio” informants claim to be treatable by animal species. The diseases cited exclusively in participant observations are signaled with.

Naturally, the MAV index also varies between 0 and 1. When close to zero it means that the species is used to treat only one or very few diseases, suggesting that its therapeutic properties are quite specific. Higher MAV indices, particularly when close or equal to one, mean that species are used to treat many different diseases, suggesting that their therapeutic properties are quite versatile, similar to those of a generic or all-purpose drug.

The UV and MAV indices can be used complementarily, since the former identifies the most popular species among a community and the latter informs about the versatility of the species, showing if they have specific/targeted or all-purpose/untargeted therapeutic properties. Notice that like the Relative Importance (RI) index of Bennett and Prance [46], the version of the UV index that we use here and the MAV index that we developed are normalized proportions of a species medicinal importance, and thus can be used in comparisons among the zootherapy practices of different traditional communities, as well as comparisons between the medicinal uses of animals and plants within and among communities.

Considering, in particular, the MAV index, it is not the first time that categories of diseases, like the ICD categories from the World Health Organization [45] or others, namely the Brazilian Center for the Classification of Diseases, are used to catalogue the descriptions given by informants (e.g., [13,47,48]). The use of such categories is useful to standardize the data shown in the literature and here we went a step further, by including them in an index that works as a measure of the species versatility in treating diseases. In this context, the MAV index is comparable to the Relative Importance (RI) index proposed by Bennett and Prance [46]. However, the indices are fundamentally different in the type of data they use. The RI index uses the specific pharmacological properties of a species (e.g., analgesic, diuretic, sedative) and the specific human body systems treated with the species derived products (e.g., skin, musculoskeletal, blood). The MAV index focuses on the categories of diseases (e.g., diabetes, asthma, rheumatism), without specifying neither for the pharmacological properties nor for the body systems treated. Both indices are relevant to measure the versatility of species in ethnomedicine, plants and animals included. The MAV index, in particular, has the advantage of using data on the categories of diseases, which are much more common in the literature and easier to collect from the field.

## Results and discussion

### The medicinal fauna at the “Riozinho do Anfrísio” Extractive Reserve

**Figure 2:**
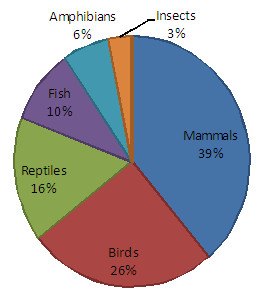
**Percentages of animal speciesused.** Percentages of animal species, by taxonomic group, used for medicinal purposes by the traditional community of the “Riozinho do Anfrísio” Extractive Reserve (Pará, Brazil).

We identified 31 animal species that are used for medicinal purposes by the “Riozinho do Anfrísio” riverine community. They are distributed in 26 families from six taxonomic groups: mammals are the most abundant (n = 12), followed by birds (n = 8), reptiles (n = 5), fish (n = 3), amphibians (n = 2) and, finally, insects (n = 1) (see Figure 2). Other studies in Brazil, in particular in Amazonia, reported similar numbers of used species for medicinal purposes: in Northeast Brazil, Alves et al. [11] identified 37 species (from 29 families) in a municipality in the state of Pernambuco; Ferreira et al. [49] recorded 29 species (17 families) in a locality in the state of Ceará; in the Midwest region, Costa-Neto and Motta [8] reported the use of 30 animal species in the Federal District; and in the North region, Branch and Silva [24] catalogued 33 species in Alter do Chão (state of Pará); Pinto and Maduro [26] catalogued 17 species in Boa Vista (Roraima state); Rodrigues [29] catalogued 29 species in Jaú National Park (Amazonas state); and Silva [33] reported 59 species in Rio Negro, also in the Amazonas state.

When considering the Amazonian biodiversity and the large numbers of medicinal plants that ethnobotanical studies usually report – often around 100 species (e.g. Rossato et al. [35] and Ragupathy et al. [50]) –, higher numbers of animal species used in zootherapy could be expected in each of the studies cited above, including in “Riozinho do Anfrísio”. Because this community is quite isolated from urban centers, lacking conventional health services, the number of species used in zootherapy should be particularly important. However, when considering species compilations for the Amazonian basin, these indicate at least 40 000 plant species, 427 mammals, 1294 birds, 378 reptiles, 427 amphibians and around 3 000 fishes [51]. The number of available plants is therefore one order of magnitude higher than that of vertebrates. The diversity of plant species to choose from is thus much higher and so it is not surprising that studies in zootherapy report a few dozen species and studies in ethnobotany report one hundred or more. Interestingly, Figueiredo [25] reported the use of an unusually high number of animal species with medicinal and magical-religious importance in the city of Belém, state of Pará. The study was, however, conducted not in a community but in the market “Feira do Ver-o-Peso”, where animal products of 73 species from various regions of the state and of the country were commercialized. At present, the trade of animal products in local markets are much more controlled by the IBAMA authorities and, consequently, some of the animal products have been disappearing from the markets and from “Feira do Ver-o-Peso”, in particular (FBB pers. obs.).

When taking into account the documented numbers of species for the “Riozinho do Anfrísio” ecoregion – the Tapajós-Xingu moist forests – these include a total of 906 species, from which 179 are mammals, 556 are birds, 58 are amphibians and 113 are reptiles (no data available on fish) [52]. The ratio is, therefore, 3:10:1:2, which is different from the ratio of medicinal uses given by the “Riozinho do Anfrísio” informants, 6:4:1:3, clearly indicating a high selectivity towards mammals. Mammals were the most representative taxon not only in our study, but in other regions of Brazil as well [8,11,18,25,26,28,33,48,53]. This pattern was also observed in other countries: in Nigeria [7,12,54], India [5,9,55,56], Mexico [10,14], Sudan [2], Bolivia [3] and Turkey [57]. In traditional Chinese medicine, mammals also play an important role, especially rhinos, tigers and bears [4]. We hypothesize that several explanations may exist for this seemingly widespread pattern: (1) the biological similarities between humans and non-human mammals; and (2) the prejudice ideas that humans sometimes have about certain non-mammal species. Also, the (3) the various ways and frequency with which people use mammals (e.g., for food and ornamentation); (4) the zoomorphism, which is the idea of transferring to humans the qualities of some animals; and (5) the high degree of knowledge that humans have about mammals, may also reinforce the medical use of these species. Nonetheless, these three last hypotheses are common to other taxa as well, particularly to birds, which are, in fact, the second group most cited by the “Riozinho do Anfrísio” informants. Considering, in particular, the zoomorphism hypothesis, the informants explained that the use of the red brocket *Mazama americana* to treat children with walking difficulties is connected to the ability of this animal to move fast. In future studies, inquiring about how many non-mammal species are known by the informants but not used in zootherapy and why, would help to better understand this appearing preference/rejection pattern for mammal/non-mammal species.

**Figure 3:**
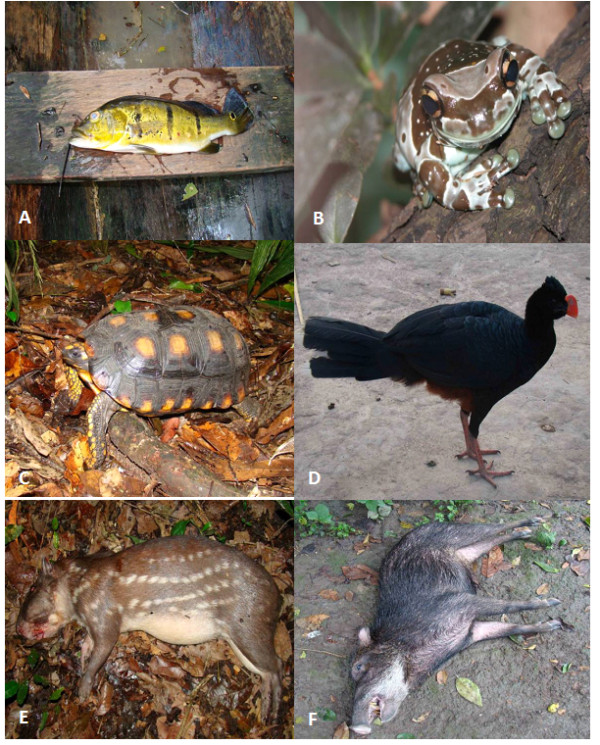
**Examples of animals usedfor medicinal purposes bythe “Riozinho do Anfrísio”community.A**) Lower Xingu Peacock *Cichla melaniae*; **B**) Rainette Kunawalu *Trachycephalus resinifictrix*; **C**) Yellow-footed Tortoise *Chelonoidis denticulata*; **D**) Razor-billed Curassow *Pauxi tuberosa*; **E**) Spotted Paca *Cuniculus paca*; **F**) White-lipped Peccary *Tayassu pecari*. Photos: **A, C, D, E** and **F** (FB Barros). Photo: **B** (MS Hoogmoed).

Of the 31 species reported in our study, seven had not yet been documented as having medicinal use in Brazil. These are the lower Xingu peacock fish *Cichla melaniae*, the grey tinamou *Tinamus tao*, the hyacinth macaw *Anodorhynchus hyacinthinus*, the blue-and-yellow macaw *Ara ararauna*, the red-and-green macaw *Ara chloropterus*, the scarlet macaw *Ara macao* and the dark-winged trumpeter *Psophia viridis* (see Table 2 for details). The uses of the razor-billed curassow *Pauxi tuberosa*[58] and of the rainette kunawalu *Trachycephalus resinifictrix*[33] were documented only recently. Ten species appear in the Appendices I, II or III of the CITES and 21 are registered at the IUCN Red List of Threatened Species (see Table 2). Figure 3 shows some examples of animals used for medicinal purposes by the “Riozinho do Anfrísio” community. All informants claimed to have used some of the above animals in medical applications and that they believe in their effectiveness.

**Table 2:** Animal species used for medicinal purposes by the “Riozinho do Anfrísio” community

**Local name**	**English name**	**Scientific taxonomy**	**Number of use reports**	**Number of informants**	**UV index**	**ICD catego- ries**	**MAV index**	**Medicinal applications**	**Body parts used (andmodes of administration)**	**UICN Red List**	**CITES Appendi- ces**	**Similarities**^**(s)**^**and differences**^**(d)**^**with other studies**
Queixada	White-lipped Peccary	*Tayasssu pecari*	22	22	0,88	10	0,10	Pneumonia♣	Teeth (1)	NT	-	[33]^(d)^
		(Mammalia: Tayassuidae)	1			11		Indigestion	Teeth (1)			
			1			10		Asthma	Testicles (2)			
Tatu-canastra	Giant Armadillo	*Priodontes maximus*	10	14	0,56	19	0,25	Snakebite	Nails (1)	VU	I	[18]^(s)^
		(Mammalia: Dasypodidae)							Fat (2, 3)			
			3			8		Earache♣	Nails (3)			
									Tail (3)			
			1			10		Asthma♣	Fat (3)			
			1			10		Cold	Fat (3)			
			1			14		Menstrual pain	Nails (1)			
			1			13		Rheumatism	Fat (3)			
Anta	Lowland Tapir	*Tapirus terrestris*	6	14	0,56	11	0,20	Indigestion♣	Fat (3)	LC	II	[33]^(s)^; [25]^(s)^; [26]^(d)^
		(Mammalia: Tapiridae)	5			14		Menstrual pain	Penis (1)			
			1			13		Hernia	Fat (3)			
			1			14		Male sexual incapacity	Penis (1)			
			1			13		Muscular pain	Nails (1)			
			1			13		Rheumatism	Fat (3)			
			1			9		Stroke	Nails (1)			
Veado	Red Brocket	*Mazama americana*	3	6	0,22	13	0,10	Difficulty of walking in children	Leg marrow (medulla) (2)	DD	-	[18]^(s)^ ; [25]^(s)^
		(Mammalia: Cervidae)	3			13		Muscular pain	Fat (3)			
			1			8		Earache	Fat (3)			
			1			13		Rheumatism	Fat (3)			
			1			12		Calluses on the feet*	Bowel fat (3)			
Caititu	Collared Peccary	*Pecari tajacu*	2	5	0,16	9	0,20	Stroke	Bowels (1)	NC	-	-
		(Mammalia: Tayassuidae)	1			10		Asthma	Testicles (2)			
			1			1		Erysipelas (“Vermelha”)	Fat (2)			
			1			13		Hernia	Fat (3)			
			1			19		Snakebite*	Fat (3)			
Tamanduá-bandeira	Giant Anteater	*Myrmecophaga tridactyla*	3	5	0,2	10	0,10	Asthma	Tail hair (1, 4)	VU	II	[3]^(d)^
		(Mammalia: Myrmecophagidae)	2			6		Spasms in children				
Paca	Spotted Paca	*Cuniculus paca*	3	4	0,12	19	0,10	Extraction of sticks and thorns of the skin♣	Bile (3)	LC	III	[18]^(s)^; [3]^(d)^; [24]^(s)^; [26]^(d)^
		(Mammalia: Cuniculidae)	1			2		Tumour	Bile (3)			
			1			4		Diabetes*	Bile (?)			
Onça	Jaguar	*Panthera onca*	3	4	0,12	10	0,05	Asthma	Fat (2)	NT	I	[33]^(s)^
		(Mammalia: Felidae)	1			13		Rheumatism*	Fat (2)			
Capivara	Capybara	*Hydrochoerus hydrochaeris*	2	2	0,08	13	0,10	Rheumatism	Bones (2)	LC	-	[33]^(s)^; [25]^(s)^; [26]^(s)^
		(Mammalia: Hydrochaeridae )	1			10		Asthma	Fat (2)			
Tatu*	Armadillo	*Dasypus* sp.	1	1	-	10	-	Asthma*	Fat (3)	?	-	-
		(Mammalia: Dasypodidae)	1			10		Cold*	Fat (3)			
			1			8		Earache*	Urine (3)			
									Tail (3)			
			1			14		Menstrual pain*	Nails (1)			
			1			13		Rheumatism*	Fat (3)			
			1			19		Snakebite*	Nails (1)			
									Fat (2)			
Coelho*	Tapeti	*Sylvilagus brasiliensis*	1	1	-	7	-	Conjunctivitis*	Faeces (2)	LC	-	[18]^(s)^
		(Mammalia: Leporidae)										
Mulher*	Woman	*Homo sapiens* (Mammalia: Hominidae)	1	1	-	18	-	Hiccup*	Milk (2)	-	-	[2]^(s)^
Mutum-fava	Razor-billed Curassow	*Pauxi tuberosa*	9	18	0,72	18	0,30	Bleeding	Beak (1)	LC	-	-
		(Aves: Cracidae)	7			19		Snakebite	Beak (1)			
			3			11		Indigestion	Beak (1)			
			2			9		Stroke	Beak (1)			
			1			4		Lack of appetite in children	Gizzard (1)			
			1			10		Pneumonia	Beak (1)			
Nambu-azulona♦	Grey Tinamou	*Tinamus tao*	16	17	0,68	19	0,10	Snakebite♣	Feet scale (1, 4)	LC	-	-
		(Aves: Tinamidae)							Feathers (1)			
			1			6		Spasms in children	Feathers (1)			
			1			19		Protection against snakebite	Feet and head (5)			
Arara azul♦	Hyacinth Macaw	*Anodorhynchus hyacinthinus*	1	1	0,04	10	0,05	Pneumonia	Beak (1)	EN	-	-
		(Aves: Psittacidae)										
Jacamim♦	Dark-winged Trumpeter	*Psophia viridis*	1	1	0,04	10	0,05	Pneumonia	Leg (1)	LC	-	-
		(Aves: Psophiidae)										
Galinha*	Domestic Chicken	*Gallus gallus domesticus*	1	1	-	8	-	Earache*♣	Fat (3)	?	-	[57]^(s)^
		(Aves: Phasianidae)	1			19		Snakebite*♣	Feathers (1)			
Arara amarela*♦	Blue-and-yellow Macaw	*Ara ararauna*	1	1	-	10	-	Pneumonia*	Beak (1)	LC	-	-
		(Aves: Psittacidae)										
Arara macao*♦	Scarlet Macaw	*Ara macao*	1	1	-	10	-	Pneumonia*	Beak (1)	LC	I	-
		(Aves: Psittacidade)										
Arara vermelha*♦	Red-and-green Macaw	*Ara chloropterus*	1	1	-	10	-	Pneumonia*	Beak (1)	LC	-	-
		(Aves: Psittacidae)										
Jabuti amarelo	Yellow-footed Tortoise	*Chelonoidis denticulata*	1	3	0,12	13	0,10	Hernia	Fat (3)	?	II	-
		(Reptilia: Testudinidae)	1			10		Pneumonia	Fat (2)			
			1			13		Rheumatism	Fat (3)			
Jacuraru	Black and white Tegu	*Tupinambis teguixin*	2	3	0,12	8	0,10	Earache♣	Fat (3)	LC	II	[18]^(s)^
		(Reptilia: Teiidae)	1			19		Snakebite	Fat (3)			
Sucuriju	Green Anaconda	*Eunectes murinus*	1	3	0,08	13	0,10	Hernia	Fat (3)	?	-	[8]^(s)^
		(Reptilia: Boidae)	1			13		Rheumatism	Fat (3)			
			1			19		Wounds	Fat (3)			
			1			9		Stroke*	Fat (2)			
Jacaré	Common Caiman	*Caiman crocodilus*	1	2	0,08	10	0,10	Pneumonia	Fat (2)	LC	I	-
		(Reptilia: Alligatoridae)	1			13		Rheumatism	Fat (3)			
Jabuti vermelho*	Red-footed Tortoise	*Chelonoidis carbonaria*	1	1	-	13	-	Hernia*	Fat (3)	?	II	-
		(Reptilia: Testudinidae)	1			10		Pneumonia *	Fat (2)			
Sapo-canuaru	Rainette Kunawalu	*Trachycephalus resinifictrix*	1	2	0,04	6	0,05	Headache	Pitch (“Breu”) (4)	LC	-	[33]^(d)^
		(Amphibia: Hylidae)	1			10		Pneumonia*	Pitch (“Breu”) (4)			
			1			11		Toothache*	Pitch (“Breu”) (4)			
Sapo-cururu*	Giant Marine Toad	*Rhinella marina* (Amphibia: Bufonidae)	1	1	-	1	-	Erysipelas (“Vermelha”)*	Abdomen (with the animal alive) (3)	LC	-	[59]^(s)^; [18]^(s)^; [24]^(s)^
			1			19		Wounds*	Entire body (toasted and macerated) (3)			
Trairão	Monster Wolf Fish	*Hoplias aimara*	1	1	0,04	8	0,05	Earache	Fat (3)	?	-	-
		(Pisces: Erythrinidae)										
Arraia*	Ray	*Potamotrygon* spp.	1	1	-	1	-	Hair lice*	Spur (6)	?	-	[33]^(d)^
		(Pisces: Potamotrygonidae)										
Tucunaré*♦	Lower Xingu Peacock Fish	*Cichla melaniae*	1	1	-	19	-	Ray sting*	Caudal fin (1)	?	-	-
		(Pisces: Cichlidae)										
Abelha*	Honey Bee	*Apis mellifera*	1	1	-	10	-	Cold*	Honey (2)	?	-	-
		(Insecta: Apidae)										

Legend: *Informations obtained exclusively from participant observations. ♦Species that were documented for the first time with medical use in Brazil. ♣The diseases and the corresponding species that were cited by the only two women that our study included: one from the semi-structured interviews and the other from the participant observations (all other species and data were cited exclusively by men). Use-reports: number of times a medicinal application was mentioned by the informants for each species. Number of informants: number of informants that cited each species for medicinal purposes. UV = species’ Use Value. ICD categories: categories of diseases described by the International Statistical Classification of Diseases and Related Health Problems of the World Health Organization (see details in Table 1). MAV = species’ Medicinal Applications Value. Modes of administration: (1) = infusion; (2) = ingestion; (3) = topical use; (4) = inhalation; (5) = amulet, (6) = mixed. Conservation categories according to the IUCN Red List: LC = Least concern; NT = Near threatened; VU = Vulnerable, EN = Endangered, CR = Critically endangered; DD = Data deficient. CITES appendices: I, II or III. (s) Studies reporting similar findings about the medicinal uses of a species; (d) studies reporting different findings about the medicinal uses of a species.

### The “Riozinho do Anfrísio” community

The use of two different methodological approaches, participant observation and semi-structured interviews, proved to be complementary, because 12 of the 31 species reported were not mentioned in the interviews, having been recorded by means of the participant observations only. This demonstrates that the more formal scenarios of the interviews are not propitious to a complete record of the animals used in zootherapy and that other methods, such as participant observations, are also important. However, to avoid conflicts in merging data from different samples, quantitative analyses were made exclusively with the data from the interviews (see the methods section for further details). Table 2, on the other hand, compiles all the data that we collected with both methods.

We found a nonsignificant correlation between the age of the informants and the number of animal species cited by them (Spearman Rank Order Correlation coefficient: r = 0.109, p = 0.605, N = 25 informants). The coefficient of determination (r^2^ = 0.012) showed that the informants’ age accounted for approximately 1.02% of the variance in the number of species cited. We expected more information from the older informants as found in other studies [60]. However, the even distribution of traditional knowledge in zootherapy among the different age groups of the studied population, suggests that the transmission of traditional knowledge between the different generations is a common practice in the “Riozinho do Anfrísio” community.

In future research, we plan to compare the information provided by men and women, since it is possible that they follow different kinds of zootherapy practices, as found in other studies [61]. Here, however, our sample from the semi-structured interviews do not allow for such comparison, because it includes one woman only – along with a second woman that provided information during the participant observations. To qualitatively distinguish the information given by these two women from that given by the men, as well as to allow future comparative studies of men versus women knowledge, we have pointed out the information given by these two women in Table 2. We decided to specifically interview the house hold heads of each family that we visited because they are the persons responsible for the dwellings and are generally assumed to be good representatives of the families’ culture, being the method employed by many ethnomedicine studies [62]. In the “Riozinho do Anfrísio”, however, household heads are mostly men, and women were generally not allowed alone with men non members of the community. Hence, to consistently interview or observe women practicing zootherapy in this community, different approaches should have to be followed, namely through the presence of women researchers in the field, which was not our case.

### The UV and MAV indices

**Figure 4:**
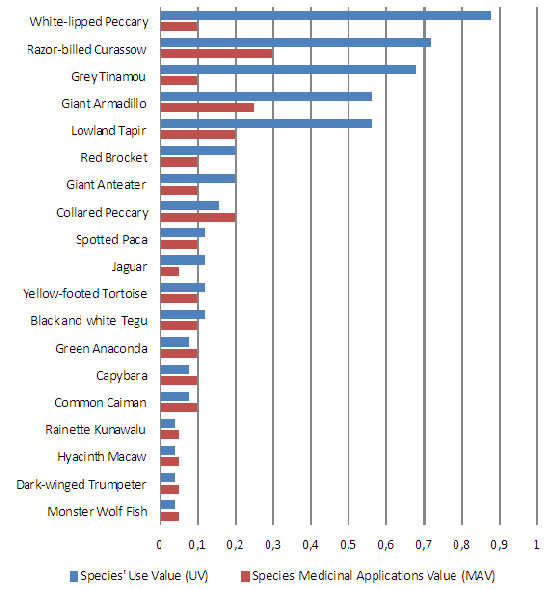
**The UV and MAVindices.** The index of Species’ Use Value (UV; N = 25 informants), adapted from Tardío and Pardo-de-Santayana [36], and the index of Species’ Medicinal Applications Value that we developed (MAV; N = 20 ICD categories of diseases and other health problems) for the 19 species mentioned in the semi-structured interviews. The Spearman Rank Order Correlation coefficient was as follows: r = 0.743, r^2^ = 0.552, p = 0.0003, N = 25 informants. See Table 2 for details on the species.

The most popular species in the “Riozinho do Anfrísio”, that is, those that were cited by more than 50% of the informants, revealing, at the same time, higher levels of agreement among them, were the white-lipped peccary *Tayassu pecari* (n = 22 informants), the razor-billed curassow *Pauxi tuberosa* (n = 18), the grey tinamou *Tinamus tao* (n = 17), the giant armadillo *Priodontes maximus* (n = 14) and the lowland tapir *Tapirus terrestris* (n = 14) (see Table 2 for details). Therefore, these are the species showing the highest Use Values: 0.56 ≤ UV ≤0.88 (see details in Figure 4 and Table 2).

Three of these five species were also among those that are used to treat more diseases (see Table 2 and Figure 4). They are the lowland tapir *Tapirus terrestris* (seven diseases within four ICD categories), the giant armadillo *Priodontes maximus* (6 within 5) and the razor-billed curassow *Pauxi tuberosa* (6 within 6). They are, therefore, the species showing the greatest Medicinal Applications Values: 0.2 ≤ MAV ≤ 0.3 (see Figure 4 and Table 2 for details). All together, the informants cited them 56 times and claimed that they treat up to 14 diseases within 9 ICD categories, that is, 45% of the 20 ICD categories of the World Health Organization. High UV and MAV indices, thus, indicate that the zootherapy knowledge about these three species is widespread and highly valued among the “Riozinho do Anfrísio” community and that their therapeutic properties may be quite effective under various contexts, which is typical of all-purpose drugs.

Accordingly, there is a positive correlation between the UV and MAV indices or, in other words, between the number of informants citing each species and the number of ICD diseases treated by them (Figure 4), with a determination coefficient of 55%. This correlation is significant despite the fact that two of the five most cited species where claimed to treat only a few diseases. Indeed, the white-lipped peccary *Tayassu pecari*, which is used to treat only three diseases among two ICD categories (MAV = 0.1), was the most cited species by the “Riozinho do Anfrísio” informants (n = 22), and the grey tinamou *Tinamus tao*, which is used to treat only two diseases among two ICD categories (MAV = 0.1), was cited 17 times (Figure 4). Hence, these are species highly valued by the population (high UV), but for which the MAV indices are among the lowest, showing that their therapeutic properties are quite specific (low MAV) instead of all-purpose.

The high UV indices of these five animal species are, therefore, informing about their level of importance to the studied community, signaling their seemingly effectiveness in treating diseases. The use of the MAV index allows a step further in this evaluation, informing about the species’ therapeutic properties: specific or targeted properties if used to treat diseases within few ICD categories (low MAV), and all-purpose or untargeted properties if used to treat diseases within several ICD categories (high MAV). Both specific and all-purpose drugs may be of interest to pharmacological research, and the use of the MAV index certainly helps to better distinguish and select among them.

### The various types of cited diseases and the animal species used to treat them

**Figure 5:**
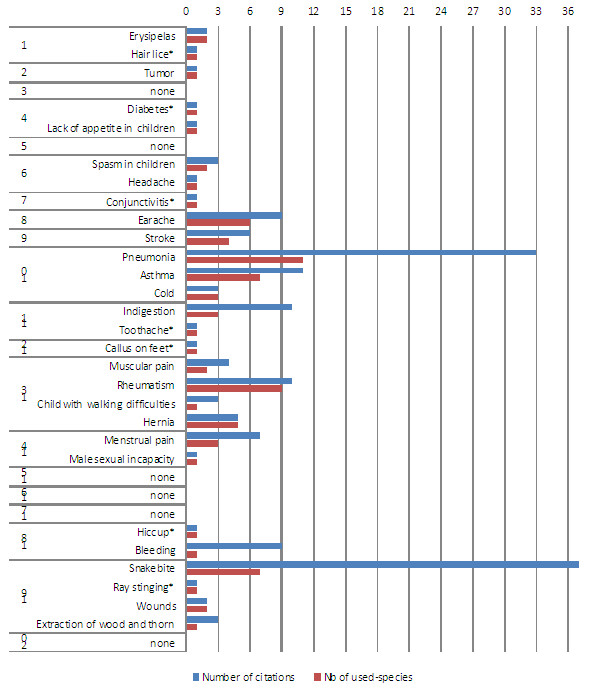
**Number of citations versusnumber of used speciesfor each disease.** Number of citations for each disease (number of times that a disease was claimed to be treated with zootherapy) and number of used species (number of species that were claimed to treat a disease). The diseases cited exclusively in participant observations are signaled with *. Correlations were made exclusively with data from the participant observations (N =21 diseases, instead of 28). The Spearman Rank Order Correlation coefficient was as follows: r = 0.803, r^2^ = 0.645, p < 0.0001, N = 25 informants. The numbers in the left axis refer to the International Statistical Classification of Diseases and Related Health Problems from the World Health Organization (ICD, 2007) [45]. See Table 1 for details.

The 31 animal species reported – qualitative data from both the interviews and the participant observations – are used for the treatment of 28 different diseases and other health problems. They are included in 14 of the 20 ICD categories of the World Health Organization [45], which means a representation of 70% (see Table 1 and Figure 5). Figure 5 shows how many categories of diseases were cited by the informants, and how many animals are used to treat them, giving an idea of the cover that zootherapy provides to the 20 ICD categories defined by modern medicine.

The most cited health problems were snakebites (n = 37 citations), pneumonia (n = 33), asthma (n = 11), indigestion (n = 10), rheumatism (n = 10), earache (n = 9) and bleeding (n = 9). These were also the diseases for which the highest number of medicinal species was mentioned. Pneumonia, for example, was assigned to 11 different species. There are two exceptions, however: indigestion and bleeding were assigned to only three and one species, respectively. Despite this, and using the data from the interviews only, we still found a significant positive correlation between the number of citations for each disease and the number of used species, with a determination coefficient of 65% (see Figure 5). In order to understand if this positive correlation has any link with the frequency with which a disease occurs or with its severity, future studies should directly inquire about the diseases that affect the most the traditional communities and the species that are used to treat them. This would contribute to a better understanding of the accumulated knowledge in zootherapy by these communities, as well as to unequivocally signal the most valued species. One exception to zootherapy in the “Riozinho do Anfrísio” is the case of malaria. It occurs in the region and affects a large number of locals, but it is not treated with zootherapy. Instead, riverines use remedies from the city to deal with it.

### Comparisons between our results and those of other studies in zootherapy

We found several similarities and differences between our data and those of other studies in other regions of Brazil and abroad (see references for some of those studies in Table 2). The same uses in distant areas of Brazil may be explained, in part, by the cultural traditions brought by immigrants from the Northeast region to the Amazonia at the time of the caoutchouc exploitation, in the late nineteenth and early twentieth centuries. Another possible explanation may be knowledge convergence between communities, based on peoples’ experience with the same diseases and animal species. For example, concerning amphibians, at “Riozinho do Anfrísio”, the giant marine toad *Rhinella marina* is used by direct contact of the abdomen of the live animal with the erysipelas (known locally as “vermelha”, meaning “red”), a type of illness that generally affects the epithelial tissue of the lower limbs. Alternatively, after being roasted and macerated, *Rhinella marina* can also be used as powder to heal wounds. Amphibians, in general, have substances on their skin that act as protection [21,63] and therefore may have antibacterial effects on erysipelas. Not surprisingly, in “Alter do Chão”, state of Pará, Branch and Silva [24] describe the use of *Bufo marinus* (= *Rhinella marina*) for scorpion bite, and also by direct contact of the skin of the animal with the wound. Barros [59] also describes the same uses of *Rhinella marina* among rural communities in the state of Paraíba, and Marques [18] documented the use of *Bufo paracnemis* (= *Rhinella schineideri*) for the same purpose in a community in the state of Alagoas. Concerning reptiles, Costa-Neto and Motta [8] reported the use of *Eunectes* sp. for rheumatism using exactly the same body parts and the modes of administration described in this study.

Mammals, the largest group in our study, deserve particular attention. Marques [18] studied the zootherapy of populations in Northeast Brazil and, as in the “Riozinho do Anfrísio”, also found the use of the deer *Mazama gouazoubira* for the treatment of children with walking difficulties, the armadillo *Dasypus novemcinctus* for earache and rheumatism, the rabbit *Sylvilagus brasiliensis* for conjunctivitis, the spotted paca *Cuniculus paca* to the removal of sticks and thorns of the skin and the black and white tegu *Tupinambis teguixin* for snakebites. In “Alter do Chão” (Pará) [24], the use of the spotted paca *Cuniculus paca* is also similar to that identified in our study, but in the Roraima region [26], the species is generally used for the treatment of stroke and cholesterol. In all three cases, the body part used is the bile. In the “Rio Negro” region, in the Amazonas state, large mammals, such as the lowland tapir *Tapirus terrestris*, the jaguar *Panthera onca* and the capybara *Hydrochoerus hydrochaeris*, are used for similar medicinal purposes as those described here [33]. For example, the capybara and the jaguar fat is recommended for the treatment of asthma. Fat is also the raw material used in most other places in Brazil [8,11,13,18,29,33], including the “Riozinho do Anfrísio” community. It is used in different ways to treat diseases. For example, the fat of the red brocket *Mazama americana*, which is used to treat muscular pain, is administered as an ointment (see Table 2 for details). In the “Feira do Ver-o-Peso” (Belém, state of Pará), Figueiredo [25] registered the commercialization of medicinal products from the lowland tapir *Tapirus terrestris*, the capybara *Hydrochoerus hydrochaeris* and the red brocket *Mazama americana*. The medicinal applications were similar to those described here. Pinto and Maduro [26], on the other hand, documented a similar use of the capybara, but a different one of the lowland tapir. An infusion of this species’ paws was recommended for the treatment of asthma. The White-lipped peccary *Tayassu peccari* provides another example of a difference: in the “Riozinho do Anfrísio” it is exclusively used to treat pneumonia, indigestion and asthma, while in “Rio Negro” it is used for the same respiratory diseases, but for uterine inflammation and malaria as well [33].

In some cases, the preparation of the drugs involves mixing animal parts with products taken from plants or other animal species. For example, at the “Riozinho do Anfrísio”, the lip of the white-lipped peccary *Tayassu pecari*, which is indicated for pneumonia, is used with cashew nuts *Anacardium occidentale* and tonka-bean seeds *Dipteryx odorata*, which, after being roasted and macerated together, are prepared and consumed as an infusion. This type of procedure has also been observed in other Brazilian studies [11] and in other countries [4,5,54]. The genitalia of several species are used in different countries as well. In “Riozinho do Anfrísio”, the penis of the lowland tapir *Tapirus terrestris* is used to treat male impotence and women’s menstrual pain. In India, Mahawar and Jaroli [56] documented the use of other animals’ penis for male impotence, as did Soewu [7] for Nigeria with the genitalia of gorilla *Gorilla gorilla* and Still [4] for China with the genitalia of rhinoceros.

“Breu” is also used in different ways. It is a viscous and aromatic substance similar to resin, found on the forest floor or in cavities in trees, which riverines attribute to the rainette kunawalu *Trachycephalus resinifictrix*, being produced during mate calls in the breeding season. This species inhabits the forest canopy at about 25 meters height and lives in hollow cavities. The “breu” is burned and wrapped in cotton so that the sick person could inhale the fragrant smoke. Among the “Rio Negro” riverines, the “breu” is inhaled and used for the treatment of strokes, epilepsy and spasms in children [33], while in the “Riozinho do Anfrísio” it is used for pneumonia, headache and toothache. Similarly to Silva [33], we could not determine, however, if the “breu” is in fact produced by the rainette kunawalu, or by the tree where the rainette nests, or if it is the result of both.

Other examples of different zootherapy practices include the spur of the ray, which is reported in “Rio Negro” for the treatment of stroke and hernia [33], but in “Riozinho do Anfrísio” is used as a comb for removing lice. While in “Riozinho do Anfrísio” the urine from the giant armadillo *Priodontes maximus* is used to treat earache, the rabbit feces *Sylvilagus brasiliensis* to treat conjunctivitis and the women milk to cure persistent hiccups, in contrast, in Sudan, woman milk is used to treat conjunctivitis [2]; in India the dog’s urine is indicated for earaches [55] and in China the tiger's urine is used to treat rheumatism [64].

Snakebites affect people worldwide, particularly those living in rural areas. In “Riozinho do Anfrísio” the riverines use body parts of various animals against snake venom, like the beak of the razor-billed curassow *Pauxi tuberosa* and the fat of the black and white tegu *Tupinambis teguixin* (see Table 2 for other examples and details). The prevalence of these incidents in the riverines’ reports may be linked to high levels of forest conservation in the area and to the types of daily activities of the riverines, which are mostly inside the forest, hence increasing the chance of encounters with snakes. The use of animal parts to treat snakebites is prevalent in many parts of the world: in Bolivia, it is treated with the bile of the spotted paca *Cuniculus paca* or with the hair of the giant anteater *Myrmecophaga tridactyla*[3]; in Nigeria [7], with the tail of the chameleon *Chamaeleo senegalensis*, the skin and jaws of the hyena *Crocuta crocuta* and the skin of the leopard *Panthera pardus*; and in Turkey, like in the “Riozinho do Anfrísio”, with the feathers of the domestic chicken *Gallus gallus domesticus*[57]. Interestingly, at the “Riozinho do Anfrísio”, snakes belong to the insect ethnocategory, which, according to the informants, includes various other animals not systematically related to insects, such as snakes, scorpions and spiders. The explanation is that all these animals can cause injuries through biting, stinging and poisoning.

Often, the use of animal parts to cure diseases is associated to popular magical-religious beliefs and practices. In “Riozinho do Anfrísio”, the collared peccary *Pecari tajacu* testicles are recommended for the treatment of asthma. They should be eaten as a meal, but the patient should not know the true origin of the medicine. Another example is the head and feet of the grey tinamou *Tinamus tao*, which, after being dried, are used as amulets to prevent snakebites. According to the riverines, this happens because this bird maintains a harmonious relationship with a venomous snake, the South American bushmaster *Lachesis muta*, building its nests in places that are often inhabited by the reptile. The magic-religious use of animal species has been observed in several studies in Brazil [6,8,11] as well as in other parts of the world [7,10,12,54]. For instance, in Mexico and in Nigeria, Jacobo-Salcedo et al. [10] reported the use of the montezuma quail *Cyrtonyx montezumae* against “evil eye”, and, in Nigeria, Sodende and Soewu [47] documented the use of the African python *Python sebae*, among other species, for protection against evil spirits.

## Conclusions

This work primarily describes the zootherapy practices of an Amazonian riverine community at the “Riozinho do Anfrísio” Extractive Reserve (state of Pará, Brazil). We interviewed 25 riverines who claimed the use of 31 animal species for the treatment of 28 diseases and other health problems, and found that seven of the species had not yet been documented with medicinal uses in Brazil. The literature available until present [19,24-29,31-33] cataloged about 140 species with zootherapy value in the Brazilian Amazonia. This number should now be revised considering the results presented here.

Using a combination of two indices to measure the medicinal importance of each species to the studied community, we were able to identify the five most popular animal species in the “Riozinho do Anfrísio” by means of the Use Value index [34-36], as well as the species’ versatility in the treatment of diseases, by means of the Medicinal Applications Value index that we developed. With this new index – comparable to the “Relative Importance” index of Bennett and Prance [46] that also measures versatility, but different in the type of data used –, we found that two of the five most popular species have specific uses, targeting diseases of four ICD categories [45], and the other three have more generic (all-purpose) uses, targeting nine ICD categories (Table 1 and Figure 4). These indices, coupled with information about the conservation status of the species (Table 2), are particularly important as they signal which medicinal animals are already at risk and those that might become threatened in the future. For example, of the five species most valued in the “Riozinho do Anfrísio”, the white-lipped peccary *Tayassu pecari* is, at present, not threatened; the razor-billed curassow *Pauxi tuberosa*, the grey tinamou *Tinamus tao* and the lowland tapir *Tapirus terrestris* are in the least concerned state; and the giant armadillo *Priodontes maximus* is vulnerable, according to the IUCN Red List (Table 2).

Hunting and fishing for food are the typical ways by which animals are caught in the “Riozinho do Anfrísio” Extractive Reserve and only a few of the listed species (Table 2) are not part of the local diet, such as the rainette kunawalu *Trachycephalus resinifictrix*, the giant marine toad *Rhinella marina*, the giant anteater *Myrmecophaga tridactyla*, the electric eel *Electrophorus electricus* and the bees *Apis mellifera*. Others are only rarely consumed, such as, the green anaconda *Eunectes murinus*, the black and white tegu *Tupinambis teguixin* and the jaguar *Panthera onca*. Besides, when capturing the animals as food, the riverines often save the body parts (e.g., bones, fat, teeth, penis, nails, beaks and feathers) or the by-products (e.g., feces, urine and milk) that have medicinal value, and often share them among neighbors and relatives. Moreover, there seems to be no commerce of the medicinal species with the urban center of Altamira, since “Riozinho do Anfrísio” is a protected area where this type of activity is forbidden. This suggests a low impact of zootherapy on the conservation status of the medicinal species used in the reserve, though we do not have this kind of data. Studies are needed that would directly address this problem [6]. However, this kind of data could be very hard to collect, since they would require long-term evaluations of the population dynamics of the used species. Alternatively, the combined use of the UV and MAV indices applied to the data that are already available in the literature on the medicinal uses of animals (and plants) in different communities of Amazonia and of other regions of the world, may be the first effective step to the identification of the species most frequently used and, by consequence, the most vulnerable. Directly inquiring about the most severe diseases, and/or the most frequent, and the species that are used to treat them, is also a necessary improvement to zootherapy studies, as this would accurately signal the most valued species by a traditional community. Moreover, given the profound knowledge that indigenous and traditional communities around the world have about their biodiversity, and the recognized importance of this kind of knowledge to biodiversity mapping and conservation (see, for example, Nazarea [65]), studies in zootherapy and ethnomedicine in general can indeed become increasingly more interesting to ecologists, as they can contribute to indicators of local biodiversity richness and species conservation status. The construction of information banks of the species used in zootherapy would thus be useful to conservation initiatives [6] – both to the animal species and the human cultures –, as well as to this purpose of mapping biodiversity.

The emergence of new health protection practices based on conventional medicine may be, however, gradually changing the traditional use of the local fauna. Some riverines from “Riozinho do Anfrísio” have reported that in recent years, particularly after the creation of the protected area, they began to have more contact with the city and access to public health services. These new dynamics may reduce their need for capturing animals for medicinal purposes only, which is positive, but may lead, at the same time, to the loss of their traditional ethnozoological knowledge and, therefore, to a decrease of their valorization of the species with medicinal and spiritual importance.

Concerning the effectiveness of the animal medicines and the positive or negative implications of its uses to human health, more studies should also be carried. Certain animal products may indeed have important therapeutic properties, but on the other hand, others may represent risks [4,8]. For instance, Still [4] draws attention to some infectious diseases (e.g., zoonoses) that can be transmitted from wild animals to humans, especially when conditions of storage and administration are precarious. Still, research in zootherapy opens the possibility of discovering new drugs, providing important benefits to society. The combined use of the UV and MAV indices as indicators of the species potential for the treatment of diseases and of their therapeutic properties, as we suggest here, is useful for the identification of the most promising species and may, therefore, accelerate the discovery of new drugs.

Interdisciplinary research, involving professionals from different fields of knowledge, may be particularly productive. Yet, the traditional knowledge of the local populations should be strictly observed. The recently approved Nagoya Protocol on the «Access to Genetic Resources and the Fair and Equitable Sharing of Benefits Arising from their Utilization», proposed by the Convention on Biological Diversity [66], opens doors for the sharing of the benefits of the drugs derived from traditional knowledge with the communities that own that knowledge. Zootherapy should be seen as a challenge for both scientists and governments in the face of its complexity, involving cultural, ethical, economical, pharmacological and conservational aspects [67].

## Competing interests

The authors declare that there are no competing interests involved in this study.

## Authors’ contributions

FBB participated in the design of the study, collected the ethnozoological data, made most of the literature survey and drafted the manuscript. SAMV performed the statistical analysis, made part of the literature survey and drafted the manuscript. HMP and LV conceived the study and participated in its design and coordination and helped to draft the manuscript. All authors read and approved the final manuscript.
